# Impaired microcirculation in the skin and subclinical atherosclerosis in individuals with dysglycaemia in a large population-based cohort

**DOI:** 10.1186/s12933-025-02628-5

**Published:** 2025-02-21

**Authors:** John Cederqvist, Karin Rådholm, Fredrik H. Nystrom, Jan Engvall, Sara Bergstrand, Ingemar Fredriksson, Tomas Strömberg, Carl Johan Östgren

**Affiliations:** 1 Department of Health, Medicine and Caring Sciences, Linköping University, Linköping, 581 83 SE Sweden; 2 Division of Diagnostics and Specialist Medicine (DISP), Linköping University, Linköping, Sweden; 3 Centre for Medical Image Science and Visualization (CMIV), Linköping University, Linköping, Sweden; 4 Department of Biomedical Engineering (IMT), Linköping University, Linköping, Sweden; 5 Perimed AB, Järfälla-Stockholm, Sweden

**Keywords:** Arterial stiffness, Coronary atherosclerosis, Type 2 diabetes, Microcirculation

## Abstract

**Background and aim:**

Dysglycaemia is a known risk factor for cardiovascular disease and microcirculatory dysfunction is associated with an increased cardiovascular disease risk. The aim of this study was to investigate the prevalence of impaired microcirculation, coronary atherosclerosis, and arterial stiffness in individuals with normo- and dysglycaemia.

**Methods:**

The study included 3,300 participants with microcirculatory measurements and information on glycaemic status, aged 50–65 years, from the Linköping site of the Swedish CArdio-Pulmonary bioImage Study (SCAPIS). Microvascular function was assessed in forearm skin using an arterial occlusion and release protocol determining peak blood oxygen saturation (OxyP). Data on pulse wave velocity (PWV) and the Coronary Artery Calcification Score (CACS) were collected. Participants were categorised into three glycaemic categories: normoglycaemia, prediabetes and diabetes.

**Results:**

OxyP was lower in the prediabetes group − 1.2%-units, 95% CI (-1.8 to -0.6) and in study participants with diabetes − 2.4%-units, 95% CI (-3.1 to -1.6) compared to the normoglycaemic group 84.3%, 95% CI (83.6 to 84.9). PWV and CACS were higher in participants with dysglycaemia. Prevalent impaired function at three vascular levels (lowest quartile of OxyP + PWV ≥ 10 m/s and CACS ≥ 100) were observed in 0.8%, 2.3% and 7.6% in the glycaemic categories respectively. The difference between the normoglycaemic and the diabetes category and the difference between the pre-diabetes and the diabetes category was significant, p = < 0.05.

**Conclusions:**

Patients with prediabetes and diabetes are more likely to have impaired microcirculation in the forearm skin and macrovascular disorders such as arterial stiffness and atherosclerosis in the coronary arteries compared to normoglycaemic individuals.

**Graphical abstract:**

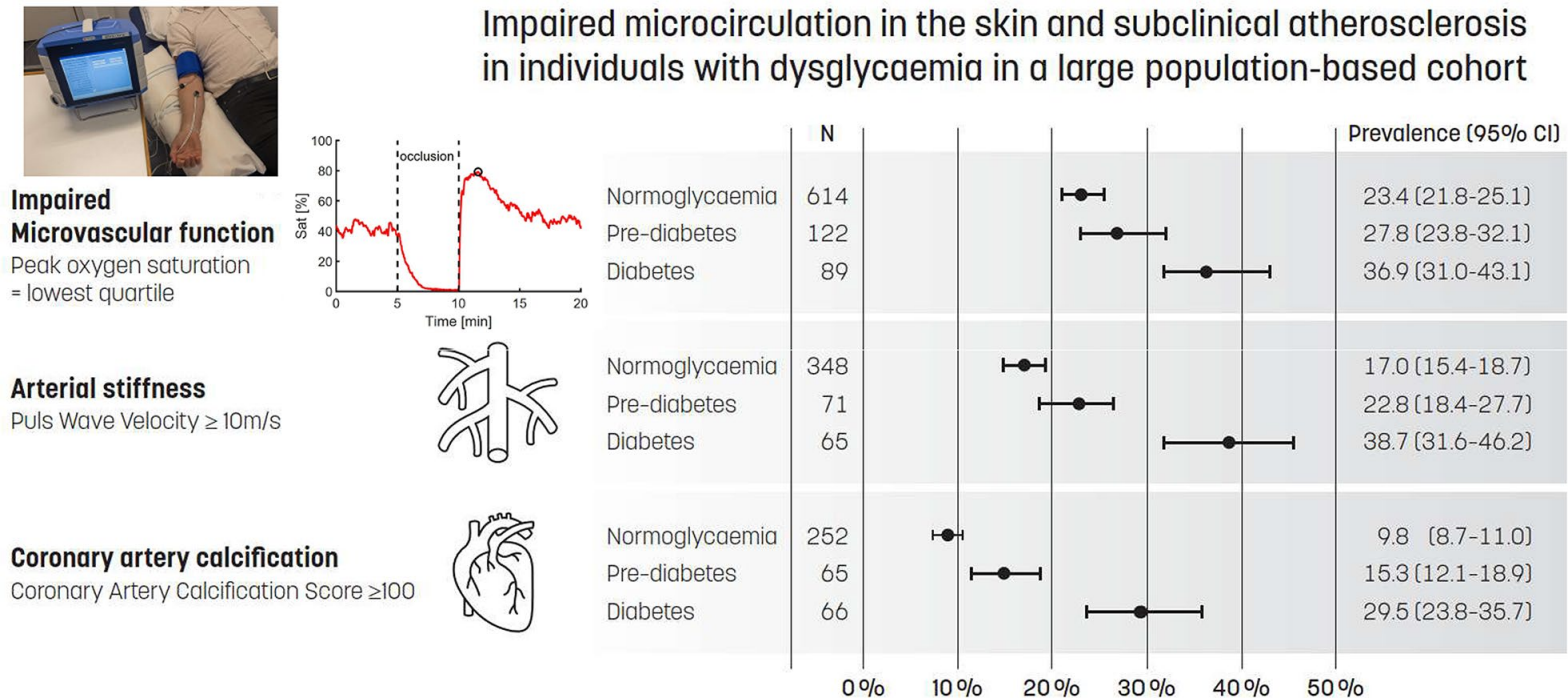

## Introduction

Diabetes mellitus and prediabetes are well known risk factors for macro-and microvascular complications such as cardiovascular disease (CVD), stroke, peripheral arterial disease and microvascular organ damage [1–6]. Type 2 diabetes (T2D) is usually preceded by a prediabetic state characterised by elevated blood glucose levels, i.e., impaired fasting glucose (IFG) or impaired glucose tolerance (IGT), both of which carry an increased risk of cardiovascular disease [7]. Although improved glycaemic control reduces the risk of macrovascular and microvascular complications, patients with diabetes are still at risk compared to the general population [8].

Microcirculatory dysfunction is associated with virtually all established cardiovascular risk factors such as type 2 diabetes, obesity, and hypertension [9, 10]. Furthermore, it is associated with systematic coronary risk evaluation [8]. In healthy individuals, impaired endothelium dependent vasodilatation and capillary recruitment is associated with increased coronary heart disease risk [11]. Microvascular reactivity is impaired in patients with acute coronary syndrome and the impairment is more pronounced in patients with acute coronary syndrome and concomitant type 2 diabetes [12]. Thus, macrocirculation and microcirculation are interconnected.

Much of the current knowledge in the microvascular field comes from small studies with experimental design in mainly disease-specific populations and there are just a few large population-based studies [13, 14].

The aim of the current study was to determine the cross-sectional prevalence of impaired microcirculation in the skin, atherosclerosis in the coronary arteries, and arterial stiffness in individuals with diabetes and prediabetes compared to normoglycaemic individuals in a large population-based cohort.

## Materials and methods

### Subjects

Data originate from the Swedish CArdioPulmonary bioImage Study (SCAPIS). The SCAPIS project, a nationwide collaboration including six Swedish universities and their affiliated university hospitals, aims to predict and prevent CVD and chronic obstructive pulmonary disease. The data collection phase in SCAPIS was carried out during 2013–18 and enrolled a total of 30,154 individuals aged 50–65. The cohort was randomly selected from the general population. The general design of SCAPIS and the baseline examinations have been previously described in detail [15, 16]. The SCAPIS study was approved by the regional ethics committee at Umeå University (Dnr 2010-228-31 M with amendment, the Swedish Ethical Review Authority, Umeå) and adheres to the declaration of Helsinki 1964 and its later revisions.

The local add-on microcirculatory examination and measurement of pulse wave velocity (PWV) were carried out at Linköping University Hospital, Linköping, Sweden, between January 2016 and June 2018. In total, microcirculation was measured in 3,809 study participants and 3,300 participants had valid microcirculatory measurements [9].

**Fig. 1:**
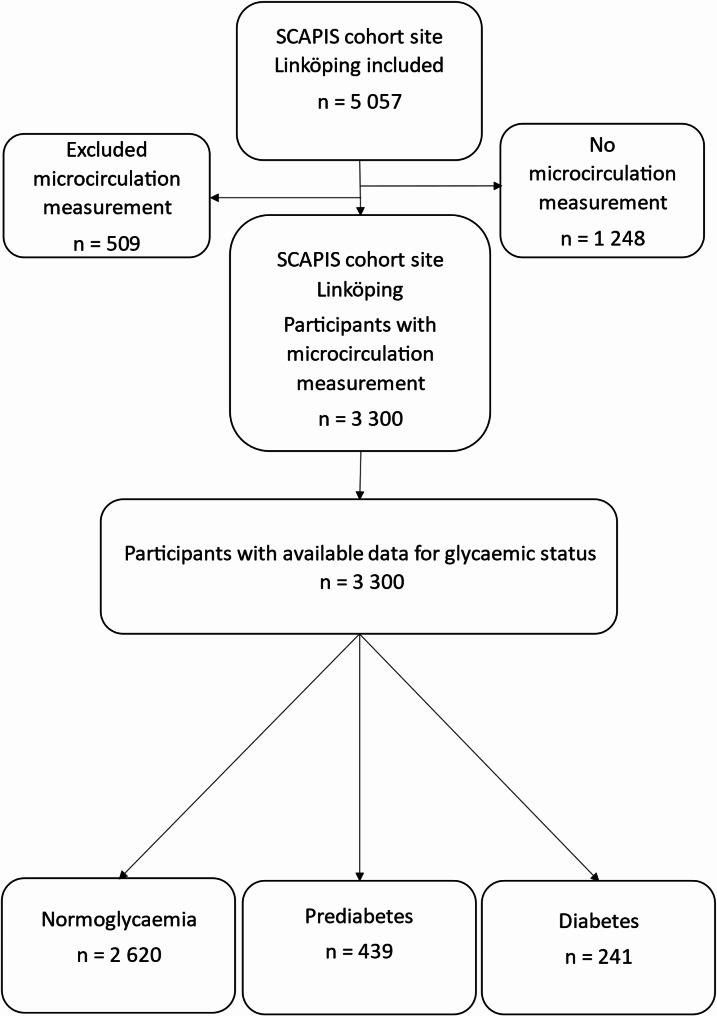
Flowchart of the study population selection process in SCAPIS Linköping

The study population selection process is illustrated in Fig. 1. The analyses of data from the local add-on studies were approved by the regional ethics committee in Linköping (Dnr 2018/156 − 31). Written informed consent was obtained from all participants.

### Glycaemic status

The study population was categorised according to glycaemic status as normoglycaemic: glucose < 6.1 mmol/L and HbA1c < 42 mmol/mol (< 6.0%), prediabetes: impaired fasting glucose: 6.1–6.9 mmol/L and/or elevated HbA1c 42–47 mmol/mol (6.0-6.4%), and diabetes mellitus: fasting glucose ≥ 7.0 mmol/L and/or HbA1c ≥ 48 mmol/mol (*≥* 6.5%) or previously known diabetes [17]. Prediabetes can be defined by either IGT, IFG or an elevated HbA1c level. In this study, we used both IFG and an elevated HbA1c value to define prediabetes [17].

### Microcirculatory evaluation

The PeriFlux 6000 EPOS system (Perimed AB, Järfälla-Stockholm, Sweden) integrates diffuse reflectance spectroscopy (DRS) and laser doppler flowmetry (LDF) with an algorithm quantifying data about the microcirculation– e.g. oxygen saturation (%), tissue fraction of red blood cells (%) and speed resolved perfusion (%RBC × mm/s) [4, 18]. The microcirculatory evaluation protocol has been described in detail previously [9].

Briefly, microvascular function was assessed in forearm skin using an arterial occlusion and release protocol determining peak blood oxygen saturation (OxyP). After a 5-min arterial occlusion the local skin blood blood oxygen saturation is near zero. The release of the occlusion creates a microcirculatory response, referred to as post-occlusive reactive hyperaemia. This response is considered an overall measure of microcirculatory function [19], which depends on many mediators such as endothelium-derived hyperpolarizing factors and sensory nerves [20], and possibly on the ability of the endothelium to induce NO-dependent vasodilation [21]. Microcirculatory OxyP is presented at post-occlusive reactive hyperaemia and was calculated as the maximum value obtained after the release of the cuff. A probe was placed to the forearm skin and microvascular parameters were measured during 5-minute baseline, 5-minute brachial arterial occlusion at 250 mmHg, and 10-minute reperfusion, to assess vascular reactivity. To standardize the microcirculatory measurements, all subjects were asked to refrain from use of nicotine, alcohol, coffee, any medication (exceptions were made for medications for: diabetes, spasticity, epilepsy, Parkinson´s disease, chronic pain and for anticoagulants and contraceptives). Prior to the examination, participants were instructed to abstain from caffeine and a heavy meal for 3 h, nicotine for 4 h and alcohol for 12 h.

### Pulse wave velocity

Sphygmocor Xcel (Atcor Medical, Australia) was used to measure pulse wave velocity. Blood pressure cuffs were placed on the left upper arm and right thigh (10–20 cm below the groin). The distance between the femoral pulse and the upper edge of the thigh cuff was subtracted from the distance between the carotid receptor and the upper edge of the thigh cuff and multiplied by 0.8. The PWV ≥ 10 m/s was used as the cut-off value for arterial stiffness.

### Coronary artery calcification

Coronary artery calcification as a marker of coronary atherosclerosis [22] was assessed in non-contrast enhanced images from a state-of-the-art multi-slice computed tomography scanner (Siemens, Somatom Definition Flash, Siemens Healthineers, Erlangen, Germany). Imaging and analyses were performed using a calcium scoring protocol and the calcium content in each coronary artery was measured and summed in the Syngo Calcium scoring software (Siemens Healthineers) to produce a total coronary artery calcification score (CACS) according to Agatston which is an international standard [23, 24]. An Agatston score ≥ 100 was defined as having coronary artery calcification.

### Statistical methods

OxyP was stratified by quartiles (Q1–Q4), with individuals with impaired microvascular function defined by the lowest quartile (OxyP Q1). In correlation analyses between OxyP vs. PWV and OxyP vs. CACS, Pearson’s and Spearman’s correlations were used. Variables with normal distribution (examined by Q-Q-plots) are presented with a mean value and standard deviation (SD). For comparing mean values between groups, we used ANOVA test for normally distributed- and a Kruskal-Wallis test for skewed variables.

It is not clear whether impaired microcirculation is caused by impaired glycaemic control or whether impaired microcirculation precedes increased blood glucose levels. Therefore, different analyses were used. Linear regression was used to examine the association between level of OxyP and glycaemic status, with OxyP being the outcome variable. Continuous variables were centered so that the intercept term in the regression equation became the expected value of the response variable when all predictors were set to their means. Centering makes the intercept more interpretable and aligns it with realistic situations, as compared to a model with non-centered variables where all continuous variables are expected to assume 0, which in this model is not biologically possible. Dummy variables were created for “smoke status” (Current and ex-smokers, never smokers for reference) and glycaemic classification (prediabetes and diabetes, normoglycaemia for reference). The intercept value, or outcome variable (beta-value), in the adjusted model is the mean value of OxyP when all categorical variables are = 0 (that is equal to their reference group) and all continuous variables are at a predicted mean value. Model 1 is adjusted for age and sex and in model 2, further possible confounders PWV, smoking status, BMI and systolic blood pressure were included in the analyses. Beta coefficients with 95% confidence intervals are given. The variance inflation factor was calculated for each independent variable in model 2 to detect signs of multicollinearity.

To evaluate whether OxyP differed between glycemic status groups, logistic regression analyses with glycaemic status as the outcome variable were performed. Binary comparisons between the three glycaemic groups and OxyP were carried out: Normoglycaemia compared to diabetes, normoglycaemia compared to prediabetes and prediabetes compared to diabetes. Results are presented as odds ratios (OR) and 95% CI. The full model was adjusted for age, sex, PWV, CACS, systolic blood pressure, smoking status, and BMI. In the analyses, OxyP, PWV and CACS values were dichotomised by OxyP Q1, increased pulse wave velocity ≥ 10 m/s and significantly increased CACS ≥ 100 in relation to glycaemic status.

## Results

Most of the study population was born in Sweden, but 217 (6.6%) individuals were born outside Sweden. The 5 most represented countries were, in descending order, Iran, Finland, Bosnia, Iraq and Poland. Baseline characteristics of the total study population and stratified by glycaemic status are shown in Table 1.

**Table 1:** Clinical characteristics of the study population by total population and stratified by glycaemic status. SCAPIS Linköping 2016–2018

	Total population	Normoglycaemia	Prediabetes	Diabetes	*p* for difference
Sample size - n	3300	2620	439	241	
**Sociodemographics**					
Women - n (%)	1639 (49.7)	1337 (51.0)	213 (48.5)	89 (36.9)	< 0.001 ^b c^
Age– mean (SD)	57.3 (4.4)	57.2 (4.4)	58.3 (4.4)	59.1 (4.1)	< 0.001 ^a b c^
**Lifestyle**					
Current smokers - n (%)	310 (9.4)	225 (8.6)	59 (13.4)	26 (10.8)	< 0.001 ^a^
**Medication**					
Blood pressure lowering agents– n (%)	588 (17.8)	369 (14,1)	104 (23.7)	115 (47.7)	< 0.001 ^a b c^
Lipid lowering agents - n (%)	228 (6.9)	100 (3.8)	40 (9.1)	88 (36.5)	< 0.001 ^a b c^
Diabetes medication– n (%)	122 (3.7)	0 (0)	0(0)	122 (50.6)	< 0.001 ^b c^
**History of cardiovascular disease**					
Previous Myocardial infarction - n (%)	50 (1.5)	26 (1.0)	12 (2.7)	12 (5.0)	< 0.001 ^a c^
Angina pectoris– n (%)	33 (1)	19 (0.7)	6 (1.4)	8 (3.3)	0.001 ^c^
**Laboratory measurements**					
eGFR– mean (SD)	81.8 (11.9)	81.8 (11.8)	82.1 (12.8)	86.0 (13.0)	< 0.001 ^b c^
HbA1c mmol/mol mean (SD)	36 (7)	34 (3)	38 (4)	54 (15)	< 0.001 ^a b c^
Total cholesterol - mean (SD)	5.6 (1.0)	5.6 (1.0)	5.3 (1.1)	4.6 (1.2)	< 0.001 ^a b c^
LDL cholesterol - mean (SD)	3.4 (0.9)	3.4 (0.9)	3.2 (0.9)	2.5 (1.0)	< 0.001 ^a b c^
HDL cholesterol - mean (SD)	1.7 (0.5)	1.7 (0.5)	1.6 (0.5)	1.4 (0.5)	< 0.001 ^a b c^
hsCRP– median (Q2-Q4), mg/L	0.9 (0.6–1.9)	0.9 (0.6–1.8)	1.3 (0.6–2.8)	1.6 (0.8–3.5)	< 0.001 ^a b c^
**Anthropometry**					
Waist Circumference - mean (SD) cm	92 (12.2)	91 (12)	97 (13)	105 (13)	< 0.001 ^a b c^
BMI, kg/m2- median (Q2-Q4), kg/m²	26.1 (23.8–28.9)	25.9 (23.6–28.4)	27.9 (25.1–31.1)	30.2 (27.3–33.0)	< 0.001 ^a b c^
**Clinical characteristics**					
Systolic blood pressure mmHg– mean (SD),	132.4 (17.6)	132 (17)	136 (18)	140 (17)	< 0.001 ^a c^
Diastolic blood pressure mmHg– mean (SD)	83.4 (10.3)	83 (10)	85 (11)	85 (11)	< 0.001 ^a c^
Pulse– mean (SD), BPM	61 (9.4)	61 (9)	64 (11)	67 (11)	< 0.001 ^a b c^
PWV - mean (SD), m/s	8.8 (1.3)	8.8 (1.3)	9.1 (1.4)	9.7 (1.4)	< 0.001 ^a b c^
PWV ≥ 10 m/s - n (%)	484 (14.7)	348 (17.0)	71 (22.8)	65 (38.7)	< 0.001 ^a b c^
PWV ≥ 10 m/s + CACS ≥ 100 - n (%)	101 (3.1)	62 (18.5)	15 (22.1)	24 (39.3)	< 0.001 ^b c^
CACS ≥ 100– n (%)	383 (11.6)	252 (9.8)	65 (15.3)	66 (29.5)	< 0.001 ^a b c^
CACS ≥ 400– n (%)	124 (3.8)	72 (2.8)	23 (5.4)	29 (12.9)	< 0.001 ^a b c^
OxyP % Q1,57.3–84.3,– n (%)	825 (25)	614 (23.4)	122 (27.8)	89 (36.9)	< 0.001 ^b c^
OxyP % Q2 84.3–87.9,– n (%)	825 (25)	624 (31.1)	131 (41.3)	70 (46.1)	< 0.001 ^a c^
OxyP % Q3 88.0–91.7,– n (%)	825 (25)	660 (25.2)	112 (25.5)	53 (22)	0.529
OxyP % Q4 91.7–101.5,– n (%)	825 (25)	722 (27.6)	74 (16.9)	29 (12)	< 0.001 ^a c^
OxyP %– mean (SD)	88.0 (5.9)	88.2 (5.9)	86.8 (6.0)	85.2 (6.0)	< 0.001 ^a b c^

Post-Hoc analysis (Significance level at < 0.05): ^a^ = Normoglycaemia vs. Prediabetes, ^b^ = Prediabetes vs. Diabetes, ^c^ = Normoglycaemia vs. Diabetes

PWV = Pulse wave velocity

CACS = Coronary Artery Calcium Sore

OxyP = Post-occlusive peak oxygen saturation

Q1 = Lowest quartile

SD = Standard deviation

m/s = meter per second

BMI = Body-mass Index

BPM = Beats per minute

The correlations between OxyP vs. PWV and OxyP vs. CACS were − 0.13 and CACS − 0.16 respectively, both *p* < 0.001.

Table 2 shows that OxyP was lower in the prediabetes group by -1.21%-units, 95% CI (-1.79 to -0.63) and in study participants with diabetes by -2.36%-units, 95% CI (-3.12 to -1.60) compared to the normoglycaemic group 84.27%, 95% CI (83.64 to 84.90). OxyP was lower in individuals with diabetes compared to individuals with normoglycemia in the fully adjusted model.

PWV and CACS increased with the severity of dysglycaemic status. Among established cardiovascular risk factors, current smoking was a predictor of microcirculatory impaired function by -3.21%-units, 95% CI (-4.0 to -2.40), *p* < 0.001. Women had a higher OxyP value than men in the fully adjusted model by 2.25%-units, 95% CI (1.77 to 2.73), *p* < 0.001. The variance inflation factor was calculated for each independent variable and no evidence of multicollinearity was found.

**Table 2:** Associations between peak oxygen saturation and glycaemic status, adjusted for confounders in two steps. Linear regression analyses with centered continuous variables were used

	Model 1 β (95% CI)	Model 2 β (95% CI)
OxyP mean (Intercept)	84.27 (83.64 to 84.90)**	85.06 (84.29 to 85.83)**
Prediabetes (ref normoglycaemia)	-1.21 (-1.79 to– 0.63)**	-0.67 (-1.38 to 0.41)
Diabetes (ref normoglycaemia)	-2.36 (-3.12 to -1.60)**	-1.26 (-2.24 to -0.28)*
Age	-0.13 (-0.18 to 0.09)**	-0.10 (-0.15 to -0.05)**
Sex (ref men)	2.58 (2.18 to 2.97)**	2.25 (1.77 to 2.73)**
Pulse wave velocity m/s		-0.06 (-0.28 to 0.16)
CACS		-0.001 (-0.002 to 0.001)
Current smokers (ref never-smokers)		-3.21 (-4.0 to -2.40)**
Ex-smokers (ref never-smokers)		-0.13 (-0.64 to 0.37)
BMI kg/m2		-0.19 (-0.25 to -0.11)**
Systolic blood pressure mmHg		-0.005 (-0.02 to 0.11)

OxyP is post-occlusive peak oxygen saturation. β-values (including intercept) are expressed as saturation in percent units (%).

*p-value < 0.05.

** p-value < 0.001.

The differences in OxyP values according to glycaemic status are shown in Table 3. The data are presented as odds ratios for a worsening of glycaemic status for each increased %-unit of OxyP. All comparisons were statistically significant when adjusted for age and sex– diabetes vs. normoglycaemia OR (95% CI) 0.94 (0.92 to 0.96), prediabetes vs. normoglycaemia OR 0.97 (0.95 to 0.98) and diabetes vs. prediabetes OR 0.97 (0.94 to 0.99). In the fully adjusted model, only the difference between the normoglycaemic category and the diabetes category remained statistically significant with OR 0.96 (0.93 to 0.99).

**Table 3:** Odds ratios (OR) for diabetes and pre-diabetes per one unit (saturation– %) increase for OxyP (post-ischemic skin peak oxygen saturation) compared to normoglycemia, and for diabetes compared to pre-diabetes. Logistic regression models were used

	Diabetes vs. normoglycaemia	Prediabetes vs. normoglycaemia	Diabetes vs. prediabetes
	OR (95% CI)	OR (95% CI)	OR (95% CI)
Crude model	0.93 (0.91 to 0.95)**	0.96 (0.95 to 0.98)**	0.96 (0.93 to 0.98)**
Adjusted for age and sex	0.94 (0.92 to 0.96)**	0.97 (0.95 to 0.98)**	0.97 (0.94 to 0.99)*
Adjusted for age, sex, PWV, CACS, systolic blood pressure, smoking status, BMI	0.96 (0.93 to 0.99)**	0.98 (0.96 to 1.00)	0.99 (0.96 to 1.02)

PWV = Pulse wave velocity

CACS = Coronary Artery Calcium Sore

BMI = Body-mass Index

^*^p-value: <0.05

^**^ p-value: <0.001

The prevalence of impaired microcirculatory function (OxyP Q1) in study participants without arterial stiffness and coronary atherosclerosis (PWV ≤ 10 m/s and/or CACS ≤ 100), showed no difference between the three glycaemic categories. The prevalence of isolated OxyP Q1 was 16.7%, 17.3% and 16.6% for the normoglycaemic, prediabetes and diabetes categories, respectively. The prevalence of impaired function in all three vascular levels combined (OxyP Q1 + PWV ≥ 10 m/s and CACS ≥ 100) were observed in 0.8%, 2.3% and 7.6% in the glycaemic categories respectively. The difference between the normoglycaemic and the diabetes category, as well as the difference between the pre-diabetes and the diabetes category was significant, p = < 0.05, is not in table.

**Fig. 2:**
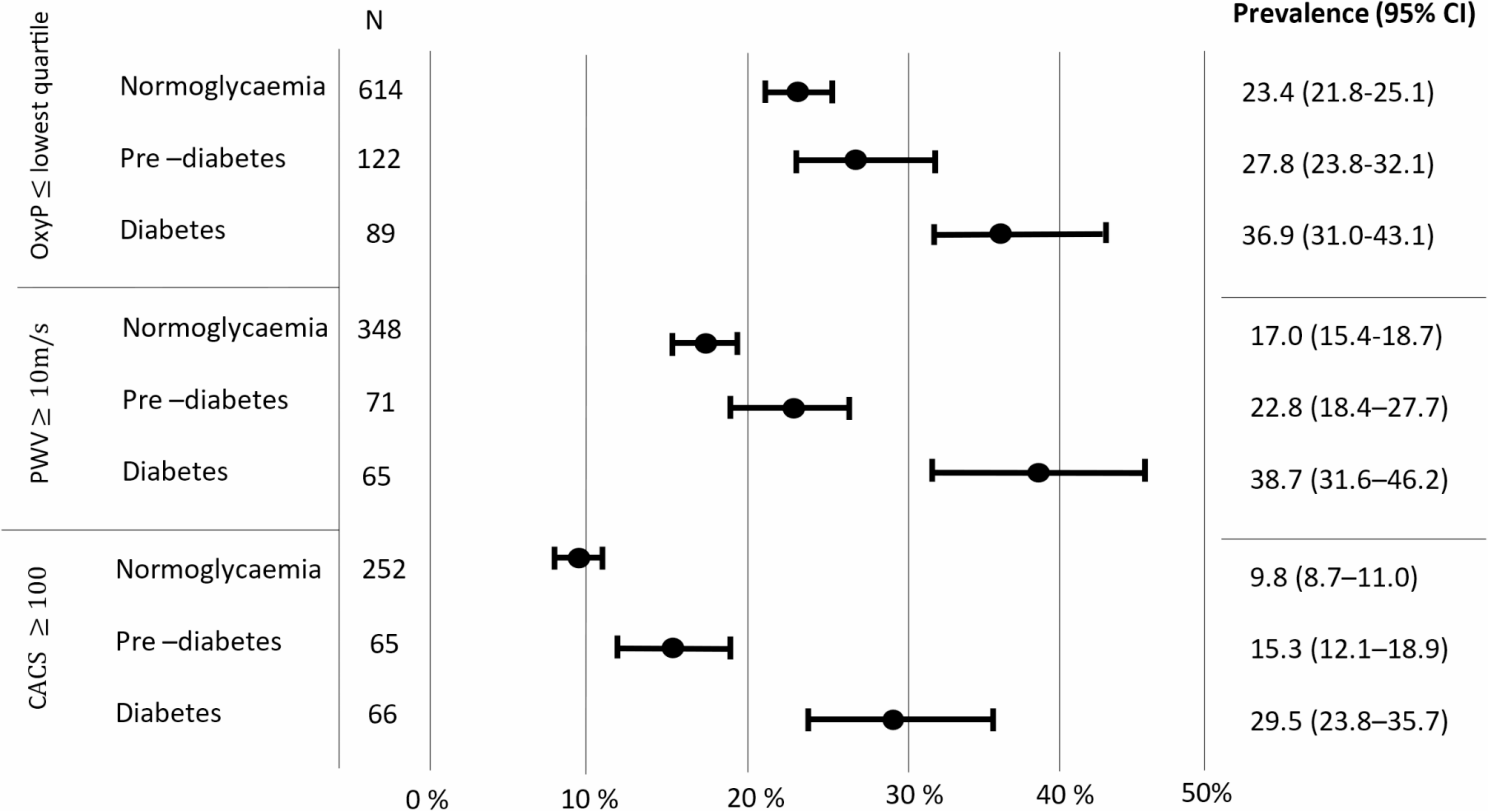
Prevalence of decreased post-occlusive skin peak oxygen saturation, increased pulse way velocity and increased coronary artery calcification in 3300 SCAPIS participants stratified by glycaemic status in three categories. OxyP post-occlusive peak oxygen saturation. PWV Pulse Wave Velocity. CACS Coronary Artery Calcium Score.

The prevalence of OxyP Q1, increased PWV (≥ 10 m/s) and CACS ≥ 100 for the study participants stratified by glycaemic status is shown in Fig. 2.

## Discussion

In this study, we investigated the prevalence of subclinical vascular disorder at three different vascular levels, i.e. impaired microcirculation in the skin, arterial stiffness and coronary atherosclerosis and their association with different stages of dysglycaemia. We found that prediabetes and diabetes, commonly referred to as dysglycaemia, were associated with increasing prevalence of impaired microcirculation in the skin, arterial stiffness and atherosclerosis in the coronary arteries. Our results expand on large previous population-based studies that related blood flow to CVD and circulatory disorders in larger vessel structures [25–27], to include different vascular beds including both macro and microcirculatory structures.

The arterial tree spanning from the large coronary artery to the minute capillaries comprises four components—elastic (conduit) arteries, muscular conduit arteries, muscular resistance arterioles and capillaries—each representing a distinct vessel system with a distinct role to play in the circulation [28]. We know that the atherosclerotic burden in the coronary and carotid arteries increases with increasing degrees of dysglycaemia [29]. In addition, the prevalence of arterial stiffness increases with worsening dysglycaemia status [30].

Impaired microcirculation is associated with established cardiovascular risk factors [9, 10] and microcirculatory changes in the retinal and renal systems have been extensively studied to understand the predictive role of glycaemic fluctuations in early diabetes [31]. Diabetic retinopathy, the leading cause of premature blindness in patients with T2D, is associated with an increased risk of cardiovascular mortality [32]. Changes in retinal micro- vessels in healthy individuals are independently associated with future risk of T2D and cardiovascular mortality [33]. Our findings suggest that microvascular dysfunction is present early in the pathophysiological processes in diabetes since OxyP was lower in the prediabetes group and in study participants with diabetes compared to the normoglycaemic group. Hyperglycemia in individuals with prediabetes as well as T2D are linked to widespread microvascular dysfunction [14]. Hyperglycemia contributes to this dysfunction through mechanisms like intraendothelial glucose accumulation, advanced glycation end products formation, oxidative stress, and low-grade inflammation [34]. Hyperinsulinemia causes vascular damage in T2D by affecting smooth muscle and endothelial cells and inducing sympathetic activation [35]. In T2D patients, small artery remodeling is characterized by hypertrophy, a pattern also seen in hypertensive patients [35, 36]. Further, emerging evidence indicates that microvascular dysfunction can precede and contribute to the onset of T2D. This dysfunction may lead to T2D through two mechanisms: impairing insulin-mediated glucose disposal and hindering insulin secretion [37].

However, controlling blood pressure reduces the onset and progression of microvascular complications in T2D patients, indicating that hypertension contributes to these complications [38]. There is growing evidence that abnormalities in microcirculation and macrocirculation are closely linked, where damage to the macrocirculation negatively impacts microcirculation, and vice versa. It is suggested that damage to small arteries caused by hypertension can be the initial step [39]. Our findings do not support this theory since OxyP was lower in individuals with diabetes compared to individuals with normoglycemia in the fully adjusted model, including both SBP, PWV and CACS. Our findings indicate that microvascular dysfunction measured as OxyP, may be a precursor to large artery and coronary calcification in individuals with T2D. However, we are not able to quantify the respective contributions of microcirculation and macrocirculation to the pathophysiological approach in T2D, which is important to explore in future research.

### Strengths and limitations

The greatest strength of the present population-based study is the sample size of 3,300 randomly selected individuals who were studied according to a common, standardised and detailed protocol for characterising vascular damage at three vascular levels and its association with dysglycaemia. However, there are some limitations. The sample sizes for the different glycaemic states are very different, with the normoglycaemic group being much larger than the diabetes and the prediabetes group. This discrepancy is inevitable in population-based studies where the prevalence of diabetes is naturally lower compared to normoglycaemia. This is different from studies that used an oversampling design, such as the Maastricht study [13, 14]. Although the smaller sample size of the diabetes group may limit the statistical power of some analyses, it provides valuable insights into the characteristics and outcomes of people with diabetes within a representative sample of the general population. Prediabetes was defined by fasting glucose level and HbA1c level as no information on glucose tolerance tests was available, we could not include these data in our definition of prediabetes. Furthermore, impaired microcirculation was defined as the lowest quartile which is not equal to dysfunction as there is no established definition of microvascular dysfunction. There is a knowledge gap in understanding the interplay of the underlying mechanisms that characterise the function of micro- and macrovascular levels and their association with prevalent atherosclerosis in coronary arteries in the general population and even more so in patients with T2D and prediabetes. It is also important to acknowledge that the microcirculatory data is derived from forearm skin. However, due to the cross-sectional design of this study, it is not possible to establish a causal relationship between microvascular function, subclinical vascular damage and dysglycaemia. Prospective studies examining microvascular and macrovascular function and coronary artery atherosclerosis could provide additional evidence of possible causal effects.

## Conclusions

We have found that patients with prediabetes and diabetes are more likely to have impaired microcirculation in the skin and macrovascular disorders such as arterial stiffness and atherosclerosis in the coronary arteries compared to normoglycaemic individuals.

This study expands on previous population-based studies to including different vascular beds; coronary circulation, large arteries and skin microcirculation. Our findings suggest that microvascular dysfunction is present early in the pathophysiological processes in diabetes. Furthermore, microvascular dysfunction measured as OxyP may be a precursor to large artery and coronary calcification in individuals with T2D.

We conclude that OxyP is a robust measure of microcirculatory impaired function and is associated with prevalent subclinical atherosclerosis and arterial stiffness.

To determine whether impaired skin microcirculation may be a useful and clinically relevant predictor of the progression of coronary artery atherosclerosis and cardiovascular disease, prospective data are needed.

## Data Availability

No datasets were generated or analysed during the current study.
